# Deposition of Gadolinia-Doped Zirconia Layers Using Metalorganic Compounds at Low Temperatures

**DOI:** 10.3390/ma14247573

**Published:** 2021-12-09

**Authors:** Agata Sawka

**Affiliations:** Department of Physical Chemistry and Modelling, Faculty of Materials Science and Ceramics, AGH University of Science and Technology, 30 Mickiewicza Av., 30-059 Krakow, Poland; asawka@agh.edu.pl

**Keywords:** gadolinia-doped zirconia layers, MOCVD method, thermal barrier coatings (TBC)

## Abstract

This paper shows the results of an investigation on the synthesis of non-porous and nanocrystalline ZrO_2_-Gd_2_O_3_ layers by metalorganic chemical vapor deposition (MOCVD) with the use of Zr(tmhd)_4_ (tetrakis(2,2,6,6-tetramethyl-3,5-heptanedionato)zirconium(IV)) and Gd(tmhd)_3_ (tris(2,2,6,6-tetramethyl-3,5-heptanedionato)gadolinium(III)). Argon and air were used as carrier gases. The molar content of Gd(tmhd)_3_ in the gas reaction mixture was as follows: 10% and 20%. The layers were synthesized on tubular substrates made of quartz glass at the temperatures of 550–700 °C. Synthesis conditions were established using the Gr_x_/Re_x_^2^ expression (Gr is the Grashof number; Re is the Reynolds number; x is the distance from the gas inflow point). The value of this criterion was below 0.01. ZrO_2_-Gd_2_O_3_ layers synthesized at 600–700 °C were crystalline. When the molar content of Gd(tmhd)_3_ in the gas reaction mixture was 10 mol.%, a relationship between the chemical composition of the gas reaction mixture and that of the deposited layer could be observed. The synthesized layers underwent scanning electron microscopy, as well as X-ray analysis. The transparency of coated and uncoated glass was tested using UV–Vis spectroscopy. Their chemical composition was examined with the use of an EDS analyzer.

## 1. Introduction

ZrO_2_-Gd_2_O_3_ system materials exhibit very promising electrical, optical, thermal, and mechanical properties. They are characterized by good ionic conductivity, which means that they can be applied as an electrolyte in solid oxide fuel cells [1,2,3,4,5,6,7,8,9,10,11]; they can emit narrow bands in the UV-B region, which influences the luminescence properties of this material [12]; and they have high radiation resistance and therefore can be used, for example, in the immobilization of nuclear waste products generated in nuclear reactor fuel [13].

Gadolinium zirconate and gadolinia-doped zirconia also seem to be promising materials for applications in thermal barrier coatings (TBCs) as a top coat [14,15,16,17,18,19,20,21,22,23,24,25,26,27,28,29,30,31,32]. Gadolinium zirconate has very low thermal conductivity, a high melting point (above 2000 °C), and its phase transformation temperatures are higher than those of yttria-stabilized zirconia [30]. Gd_2_Zr_2_O_7_ is stable at 1500–1550 °C [17,25,28]. It is also characterized by excellent chemical resistance to CaO-MgO-Al_2_O_3_-SiO_2_ (CMAS) attack [17,20,22,26,28,30]. Its disadvantage is, however, a reduced fracture toughness, which leads to easy crack formation and lower erosion resistance [25,26]. However, it seems [32] that the addition of YbSZ (Yb_2_O_3_-stabilized ZrO_2_) to Gd_2_Zr_2_O_7_ could improve its fracture toughness. Gd_2_Zr_2_O_7_ coatings for TBC applications are deposited mainly using methods such as atmospheric plasma spraying (APS) [16,22,27], electron beam physical vapor deposition (EB PVD), [19,24,26,28,30], or suspension plasma spraying (SPS) [20]. Attempts to obtain Gd_2_Zr_2_O_7_ coatings were undertaken using the metalorganic chemical vapor deposition (MOCVD) method [17,18]. The manufacturing and the properties of Gd_2_Zr_2_O_7_ coatings have been studied extensively for many years; however, investigations on the synthesis of Gd_2_O_3_-doped ZrO_2_ layers have been significantly less developed.

Gd_2_O_3_ is considered an alternative stabilizer to Y_2_O_3_ in ZrO_2_-based materials applied in TBCs as a top coat. Yttria-stabilized zirconia is conventionally used in TBCs. There are three polymorphic phases of ZrO_2_: monoclinic, tetragonal, and cubic. The addition of 7 to 8 wt.% (4 to 4.5 mol.%) Y_2_O_3_ (4YSZ) allows for the stabilization of the tetragonal prime (t’) phase. This is the most advantageous phase for these applications due to its low thermal conductivity and high fracture toughness. The use of the t′-ZrO_2_ phase in TBCs can produce a longer thermal cycle life than is possible in the case of the cubic phase [31,33]. However, it undergoes decomposition into high and low yttria phases upon prolonged exposure at elevated temperatures. After cooling, these second phases transform into the monoclinic phase, which is accompanied by an increase in its volume and can lead to the destruction of TBCs. Their acceptable application temperature is 1200 °C. The next limitation of their use is the sintering process, which can lead to a reduction of their high-temperature capability and a loss of their strain tolerance [33,34].

In a study [14], Gd_2_O_3_-stabilized ZrO_2_ coatings were obtained using plasma spraying (PS). The addition of 4 mol.% of Gd_2_O_3_ led to a slower sintering process of Gd_2_O_3_-stabilized ZrO_2_ top coat and a lower thermal conductivity than Y_2_O_3_-stabilized ZrO_2_. However, it had a lower resistance to the destabilization of the metastable tetragonal (t’) phase than yttria-stabilized zirconia. It appears that further research is needed to solve this problem.

The aim of this study was to obtain non-porous, nanocrystalline, Gd_2_O_3_-doped ZrO_2_ layers using MOCVD. The presence of pores in materials reduces their thermal conductivity but, on the other hand, acts as stress concentrators in materials. Moreover, pores allow for the easy penetration of CMAS into the substrate. Hence, they are characterized by low resistance to thermal stresses. Their non-porous and nanocrystalline microstructure should assure their low thermal conductivity, as well as high mechanical strength. Investigations on the influence of nanostructured microstructure of a top coat on TBC properties were conducted for yttria-stabilized zirconia coatings [34]. It was found that the nanocrystalline coatings of yttria-stabilized zirconia could significantly improve the performance of TBCs. It is thought that nanostructured yttria-stabilized zirconia exhibits excellent thermal insulation capability and durability at high temperatures. The reduced thermal conductivity is a result of the scattering of phonons at the grain boundaries of nanocrystalline material. Thus, the coatings could be very thin, ensuring low component weight. It was also observed that the thermal conductivity of nanocrystalline yttria-stabilized zirconia coating was about 15% lower than in the case of microcrystalline material. Investigation into the resistance to thermal shocks of nanocrystalline Y_2_O_3_-stabilized ZrO_2_ coatings demonstrated that the number of thermal shock cycles due to material failure was about two to three times higher than for conventional microcrystalline coating. It was also noted [21] that nanostructured yttria-stabilized zirconia coatings demonstrated better resistance against oxidation and hot corrosion than conventional ones. Moreover, nanocrystalline coatings contained fewer microcracks. It should be mentioned that nanocrystalline yttria-stabilized zirconia coatings have been deposited by the APS method [21,34], and coatings manufactured with its usage are usually characterized by high porosity and low mechanical strength. Such studies have not been carried out in the case of Gd_2_O_3_-stabilzed ZrO_2_ synthesized by MOCVD. However, it seems that the deposition of non-porous and nanocrystalline Gd_2_O_3_-stabilized ZrO_2_ layers with the use of this technique should also be advantageous and significantly improve the performance of TBCs.

This paper presents the results of research on the microstructure, structure, and chemical composition of Gd_2_O_3_-doped ZrO_2_ layers synthesized by MOCVD onto substrates of complex shape.

## 2. Materials and Methods

Gd_2_O_3_-doped ZrO_2_ layers were obtained by the MOCVD method with the use of Zr(tmhd)_4_ (tetrakis(2,2,6,6-tetramethyl-3,5-heptanedionato)zirconium(IV)) (Aldrich, Saint Louis, MO, USA) and Gd(tmhd)3 (tris(2,2,6,6-tetramethyl-3,5-heptanedionato)gadolinium(III)) (Alfa Aesar, Haverhill, MA, USA) as reactants. Argon, as well as air, were used as carrier gases. For carbon elimination during the layer growth, air was used.

The deposition temperature was in the range of 550–700 °C. Pressure was in the range of 5 × 10^2^–6 × 10^3^ Pa. The deposition time was 10–30 min. The evaporation temperature of Zr(tmhd)_4_ was changed from 260 to 280 °C, and in the case of Gd(tmhd)_3_, from 170 to 190 °C. Reactants were weighed and the amounts were chosen to ensure their appropriate molar ratio. Due to differences in their evaporation temperatures, a special original evaporator was designed, which was placed in the furnace with gradient temperature distribution. This ensured an appropriate evaporation temperature for each of the reactants. This solution ensured a constant molar ratio of reactants. Their vapors were transported to the CVD (chemical vapor deposition) reactor by pure argon (the molar ratio of reactants was constant). The molar content of Gd(tmhd)_3_ in the gas reaction mixture was 10% or 20%.

The magnitude of the Gr_x_/Re_x_^2^ expression was less than 0.01 (Gr is the Grashof number; Re is the Reynolds number; x is the distance from the gas inflow point) [35,36,37]. The use of this criterion is helpful in the synthesis of dense and uniform-thickness layers. The deposition parameters selected using this expression allow for the prevention of the homogeneous nucleation process during layer growth. It includes factors such as chemical reactions, heating of cold gases by a hot substrate, irregular shape of the reactor, and mode of gas waste removal. Low Gr_x_/Re_x_^2^ values favor elimination of the homogeneous nucleation, and allow the production of uniformly thick layers on large substrates with complicated shapes [35]. A diagram of MOCVD equipment is shown in [38].

ZrO_2_-Gd_2_O_3_ layers were deposited on the inner surfaces of tubular substrates. Commercial tubes were made of quartz glass (Φ_external_ = 14 mm, Φ_internal_ = 13 mm, and L = 25 mm). Quartz glass substrates were used as substitute substrates. The main advantages of their use are the easy observation and assessment of the obtained layers due to the transparency of the substrates, and their low cost [36,37,39].

Surfaces and cross-sections of synthesized layers were examined with the use of a scanning electron microscope (SEM NANO NOVA 200) produced by FEI EUROPE COMPANY (Eindhoven, The Netherlands), and an energy dispersive X-ray spectroscope (EDS) microanalyzer manufactured by EDAX EDS Company (Mahwah, NJ, USA). X-ray diffraction analyses were performed with the use of an X’Pert X-ray diffractometer from Panalytical (Malvern, UK). The transparency tests of coated and uncoated glass were carried out using a UV–Vis Spectrophotometer JASCO V630 from JASCO Deutschland GmbH (Pfungstadt, Germany).

## 3. Results and Discussion

Deposited layers were visually transparent and without white powders. This means that during layer growth, homogeneous nucleation did not occur. Products of homogeneous nucleation are formed in the gas phase and are in the form of porous solid particles. Larger particles settling on the deposited layers disturb their microstructure and deteriorate their properties. Furthermore, the layers are characterized by poor adhesion to the substrate and by lower mechanical strength and corrosive resistance. Their presence reduces their transparency when the deposited layers are amorphous.

**Figure 1:**
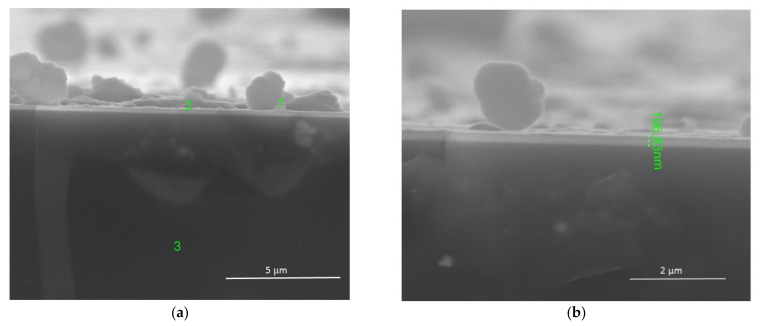
ZrO_2_-Gd_2_O_3_ layer deposited at 550 °C (surface and cross-section) (**a**) and its thickness (**b**). Molar content of Gd(tmhd)_3_ in the gas reaction mixture: 10%. Synthesis time: 30 min.

Figure 1 presents an example of a ZrO_2_-Gd_2_O_3_ layer obtained at a temperature of 550 °C. The molar content of Gd(tmhd)_3_ in the gas reaction mixture was 10%. The results from Figure 1b show that the layer was non-porous and uniform in thickness (about 200 nm). The layer growth rate was about 400 nm/h. EDS point analysis was performed at two different points of the layer (Figure 1a confirms the presence of Zr and Gd in the layer (Table 1)).

Calculations of the molar content of Gd_2_O_3_ in the layers indicated that there was a relationship between the chemical composition of the gas reaction mixture used and the composition of the layer deposited. If the molar content of Gd(tmhd)_3_ in the gas reaction mixture was 10%, the content of Gd_2_O_3_ in the layer should amount to about 5 mol.%. By analyzing the data in Table 1, it seems that these results are in line with expectations. This also means that the process of the layer deposition was controlled by the diffusion of reactants to the substrate. Subsequently, the value of the fraction of substrate (layer) surface available for the adsorption of reactants and solid reaction products (1-Θ_z_, where Θ_z_ is the surface fraction, where there is adsorption) may be large, and the reactant concentration p_i(s)_ on the substrate surface is close to zero [35,40]. When the synthesis is controlled by the surface reaction rate, then the concentration of the reactants used on the substrate p_i(s)_ is greater than zero and the reactant adsorption can be more intensive. Hence, competition in adsorption can occur between these reactants. For this reason, there is a difference between the molar contents of reactants supplied to the CVD (MOCVD) reactor and those adsorbed on the substrate surface [35,40].

**Figure 2:**
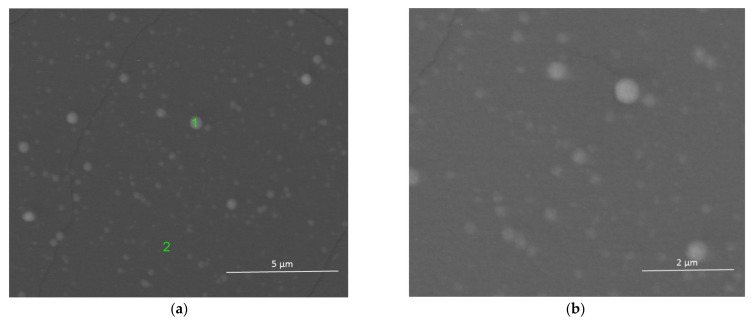
ZrO_2_-Gd_2_O_3_ layer deposited at 550 °C. Magnification: ×20,000 (**a**) and magnification: ×40,000 (**b**). Molar content of Gd(tmhd)_3_ in the gas reaction mixture: 20%. Synthesis time: 30 min.

The surface of the layer was also synthesized at 550 °C, but with a higher molar content of Gd(tmhd)_3_, i.e., 20% as shown in Figure 2.

The layer was also non-porous and contained Zr and Gd (Table 2).

EDS point analysis at point 1 (brighter surface) and at point 2 (darker surface) also allowed us to ascertain that, especially in the case of point 1, the amount of dopant in the layer was overstated (it should be about 10 mol.%, but was about 15 mol.%). At point 2, this value amounted to about 12 mol.%. Most likely, the process of the layer deposition was controlled by the surface reaction rate. There was a large differentiation in the reactant adsorption on the substrate as a function of the temperature and the reactant concentration. The use of a high dilution of the reactants in the carrier gases, and/or the reduction of the diffusion coefficients of reactants, can cause a decrease in the transition temperature from the synthesis controlled by the reaction rate to that controlled by mass diffusion to the substrate from the gas phase.

**Figure 3:**
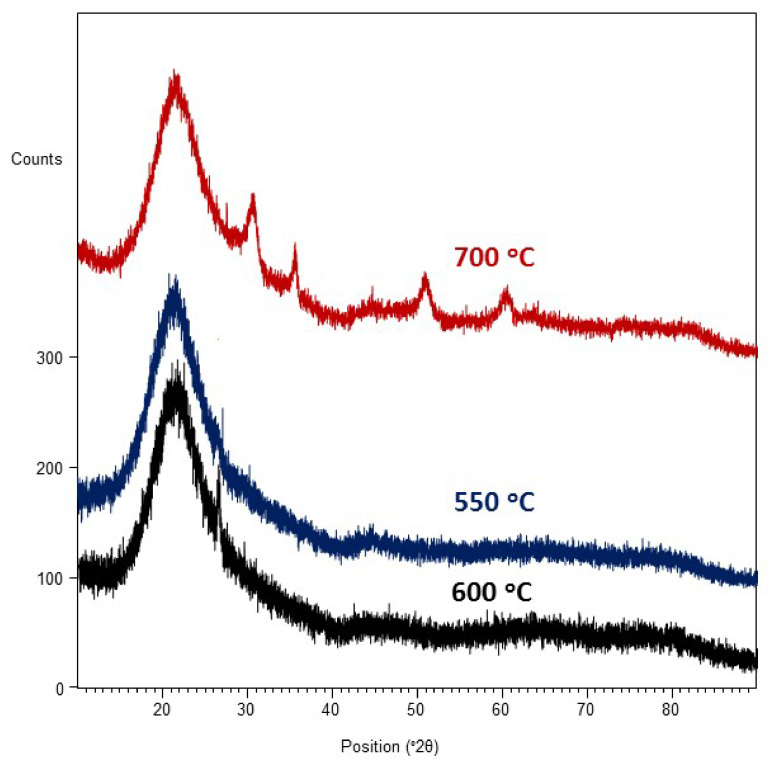
X-ray diffraction pattern of ZrO_2_-Gd_2_O_3_ layers deposited at 550, 600, and 700 °C. Molar content of Gd(tmhd)_3_ in the gas reaction mixture: 10%.

It is also supposed that the difference in the molar contents of dopant in the layer was likely a result of the occurrence of Zr^4+^ ion segregation; Zr^4+^ has a smaller ionic radius than Gd^3+^ ions (the ionic radius of Zr^4+^ is 0.84 Å and that of Gd^3+^ is 0.934 Å). Their segregation takes place from more ordered areas (brighter surface) to less ordered areas (darker surface). A similar phenomenon was observed in [41]. Furthermore, the layer likely includes aggregates consisting of nanocrystallites. However, X-ray analysis (Figure 3) showed that the deposited layer was amorphous.

It should also be noted that the layers were synthesized on commercial substrates. Due to the presence of unmelted glass sand in quartz glass, the substrate surface was not smooth. Defects on a glass surface are in the form of convexities. The coordination number of atoms on these surface parts is smaller. Hence, more intensive adsorption of reactants can occur there than on planar parts of the glass substrate. At higher temperatures, it can also be the reason for the presence of a greater number of grain forms in the layer.

**Figure 4:**
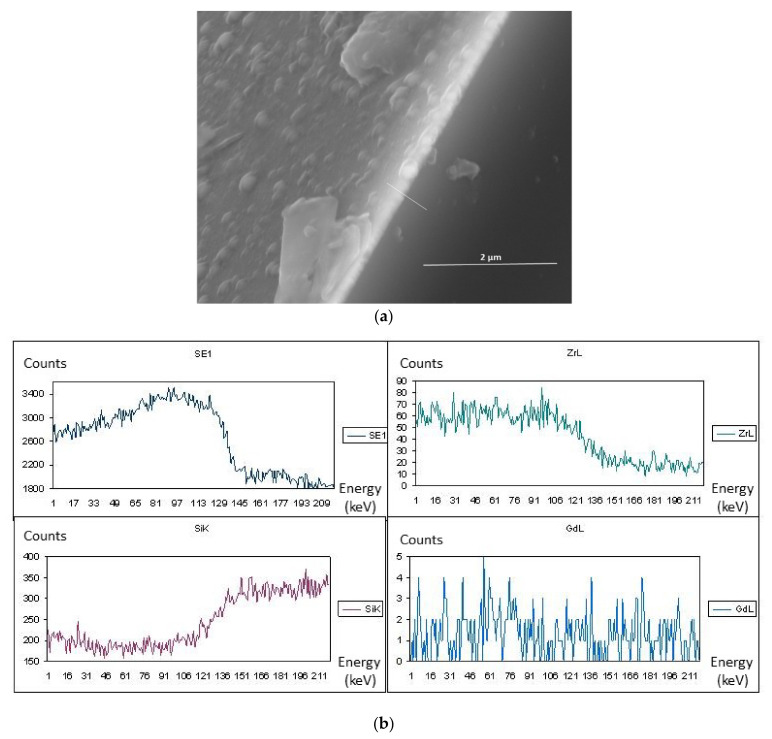
ZrO_2_-Gd_2_O_3_ layer deposited at 600 °C (its surface and cross-section) (**a**), with linear EDS analysis (**b**). Molar content of Gd(tmhd)_3_ in the gas reaction mixture: 10%. Synthesis time: 30 min.

In Figure 4a, the results show that the layer deposited was also non-porous and uniform in thickness. The layer was synthesized at a temperature of 600 °C with 10 mol.% content of Gd(tmhd)_3_. It seems that the beginnings of its crystallization were already visible (Figure 3). Numerous aggregates were also present on its surface (Figure 4a). From the linear EDS analysis (Figure 4b), the results showed that the layer contained Zr as well as Gd.

**Figure 5:**
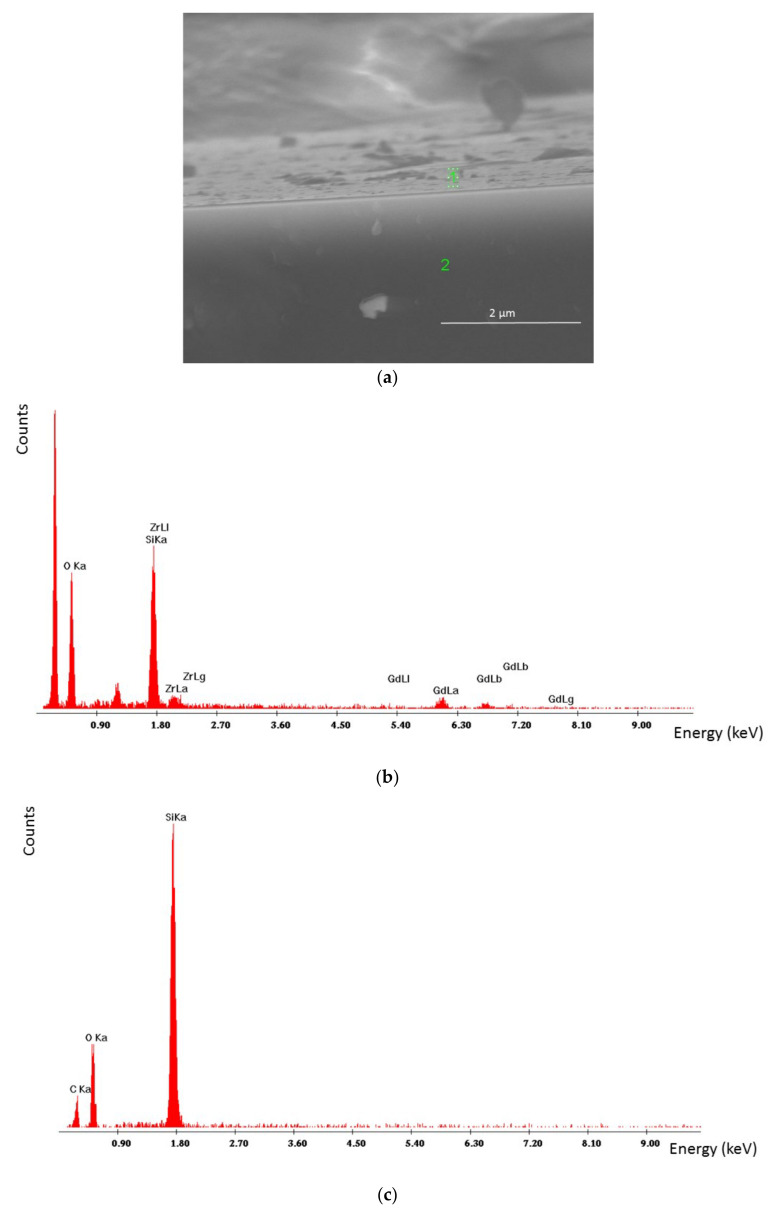
ZrO_2_-Gd_2_O_3_ layer deposited at 600 °C (its surface and cross-section) (**a**), with EDS analysis at point 1 (**b**) and point 2 (**c**). Molar content of Gd(tmhd)_3_ in the gas reaction mixture: 20%. Synthesis time: 15 min.

Figure 5 shows the surface and the cross-section of the layer deposited at the same temperature, but when the molar content of Gd(tmhd)_3_ was 20%. The synthesis time was 15 min. The deposited layer had no pores and was uniform in thickness (Figure 5a). EDS analysis confirmed the presence of Zr and Gd in the obtained layer (Figure 5b). Most likely, the presence of carbon in the layer (Figure 5c) was due to the fact that the samples had to be sputtered with carbon before SEM and EDS tests. Despite the layer being thinner (the synthesis time was twice as short), numerous aggregates were visible on its surface.

**Figure 6:**
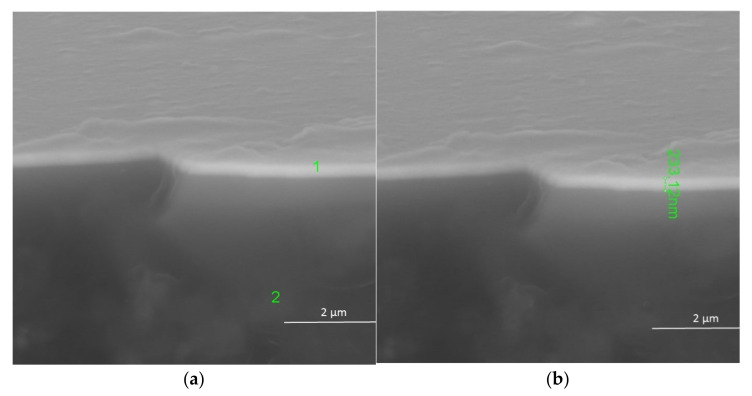
ZrO_2_-Gd_2_O_3_ layer deposited at 700 °C (its surface and cross-section) (**a**) and its thickness (**b**). Molar content of Gd(tmhd)_3_ in the gas reaction mixture: 10%. Synthesis time was 30 min.

In Figure 6, the surface and cross-section of the layer, deposited at 700 °C are shown. The layer had a thickness of about 235 nm, was without pores, and was uniform (Figure 6b). The layer growth rate was about 470 nm/h. This growth rate is slightly different from that of the layers deposited at a temperature of 550 °C.

The results of the EDS point analysis carried out at point 1 (Figure 6a) are in alignment with the expectations for quality and quantity (Table 3).

It is possible that the process of the layer synthesis was controlled by mass diffusion to the substrate. This also means that the chemical composition of the layers deposited could be controlled under these synthesis conditions. In this case, the concentration of the reactants on the substrate surface was lower, and competition in the adsorption between both reactants did not occur. This competition depends on the concentration of the reactant used in the gas phase and the substrate temperature. The Figure 3 results show that the layer was crystalline (Figure 3). This was a solid solution of Gd_2_O_3_ in ZrO_2_.

**Figure 7:**
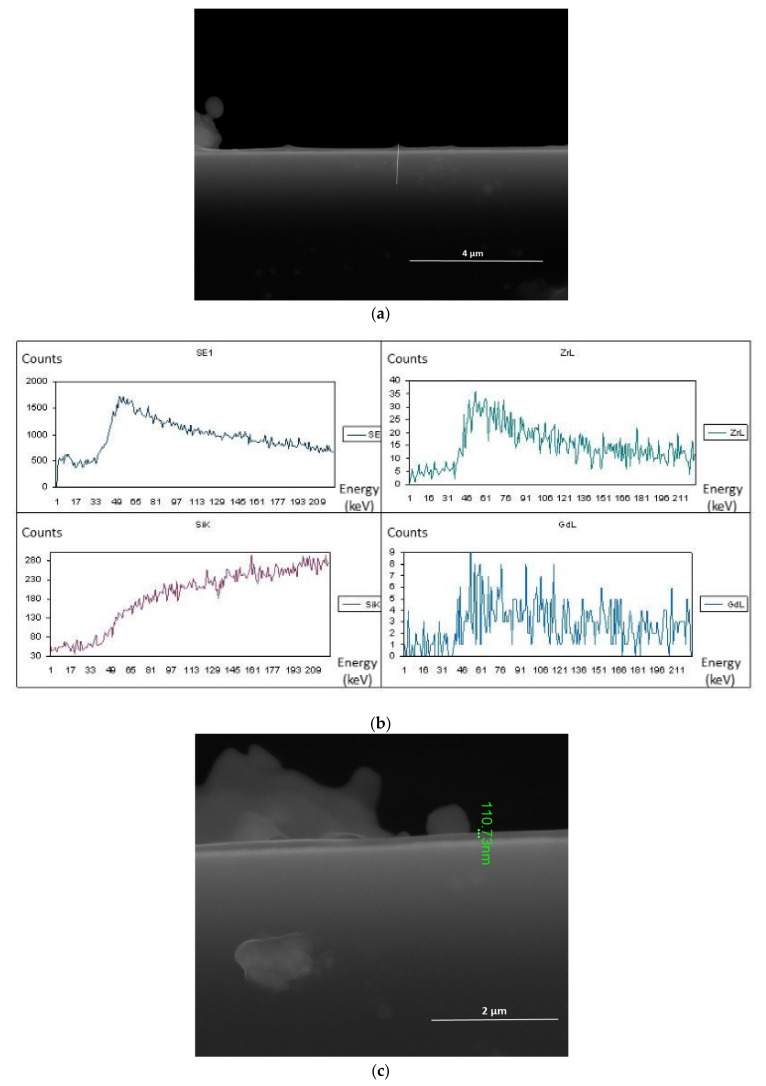
ZrO_2_-Gd_2_O_3_ layer deposited at 700 °C (**a**) and its cross-section (**c**) with linear EDS analysis (**b**). Molar content of Gd(tmhd)_3_ in the gas reaction mixture: 20%. Synthesis time: 15 min.

A cross-section of the layer obtained at 700 °C, but with 20 mol.% content of Gd(tmhd)_3_ in the reaction mixture, is presented in Figure 7. However, in this case, the synthesis time was shorter than previous ones; it was 15 min. The layer thickness was about 111 nm (Figure 7c). In this case, the layer growth rate was about 440 nm/h. This finding confirmed that the layer growth rate was constant. The layer growth rate can be independent of the temperature, when other synthesis conditions are appropriately selected. As mentioned, the synthesis conditions were established so that Gr_x_/Re_x_^2^ did not exceed 0.01 [35,36,37]. If the synthesis temperature is higher, the reactant concentration should be lower. The amount of reactants would remain the same, but the amount of diluent gas (e.g., argon) should be higher.

From linear EDS analysis (Figure 7b), it can be seen that Zr and Gd were present in the obtained layer.

**Figure 8:**
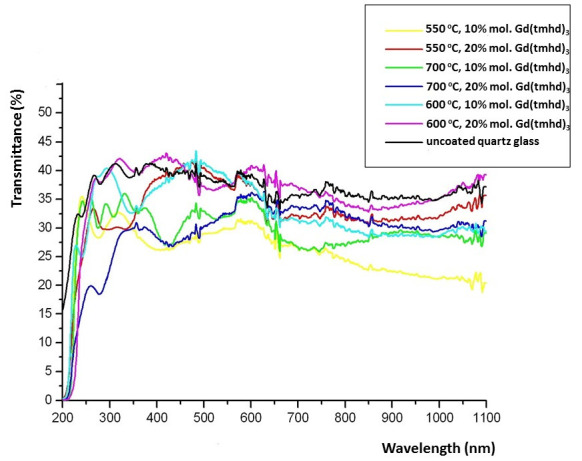
Transparency of the quartz glass covered with ZrO_2_-Gd_2_O_3_ layers deposited under various conditions and uncoated glass.

In order to check whether the homogeneous nucleation process was present during the layer growth, the transparency tests of coated and uncoated substrates were performed (Figure 8). In Figure 8, the results show that the transparency of amorphous layers was similar to that of uncoated quartz glass. When the layers became crystalline, their transparency decreased. This appears to be a consequence of light scattering at the grain boundaries. A similar situation was observed in other studies [37,40]. However, when the layer was obtained at 550 °C with 10 mol.% content of Gd(tmhd)_3_ in the gas reaction mixture (yellow line), a decrease in its transparency was observed above about 750 nm. As mentioned, the layers were synthesized on commercial tubes made of quartz glass and were synthesized on their inner surfaces. The curved shape of the substrates was a reason for measurement difficulties. It should also be noted that this commercial glass showed some roughness that was caused by the presence of unmelted sand fragments on its surface. The layers are able to be deposited easier on the unevenness of the substrate due to a higher energy of the convex surface (coordination number of atoms on this surface is subsequently lower). On its uneven surface, more intensive adsorption of reactants takes place, and the layer crystallization process can more easily occur than on flat parts of the substrate. As a consequence, light scattering can easily occur. Surfaces of commercial substrates can differ from each other. Hence, in this case, the transparency of the sample could be reduced despite the layer being amorphous. If a homogeneous nucleation process is present during the layer growth, the layer transparency would be reduced significantly.

## 4. Conclusions

The presented results of our investigation into the deposition of ZrO_2_-Gd_2_O_3_ layers on the tubes made of quartz glass with the use of MOCVD showed that non-porous amorphous or crystalline layers can be deposited at low temperatures, i.e., 550–700 °C. They are also uniform in thickness. ZrO_2_-Gd_2_O_3_ layers synthesized at temperatures of 600–700 °C exhibit crystallinity. When the synthesis temperature was lower and the molar content of Gd(tmhd)_3_ in the gas reaction mixture was higher (20 mol.%), the segregation of Zr^4+^ ions in the layers could be observed due to their smaller ionic radius compared to that of Gd^3+^ ions. As a consequence, the amount of Gd_2_O_3_ in the layer was different than expected. When the molar content of Gd(tmhd)_3_ in the gas reaction mixture was lower (10 mol.%), there was a relationship between the composition of the gas reaction mixture used and that of the layers synthesized at temperatures of 550 and 700 °C. It seems that the synthesis was also subsequently controlled by mass diffusion to the substrate. It is important to determine the transition temperature from the deposition controlled by the reaction rate on the substrate to that controlled by mass diffusion to the substrate from the gas phase. This temperature can be significantly reduced when the reactants are highly diluted in the carrier gases.

At lower temperatures, the presence of aggregates was observed, irregularly distributed over the surface. When the synthesis temperature was higher, their numbers became higher and were more regularly distributed.

UV–Vis testing confirmed that homogeneous nucleation did not occur during the layer deposition. When the layer crystallization process became more advanced, a decrease in its transparency could be observed.

## Figures and Tables

**Table 1:** Average chemical composition carried out by EDS point analysis at points 1 and 2 presented in Figure 1a.

Point Number	Element	Weight (%)	Atomic (%)	Expected Molar Content of Gd_2_O_3_ in the Layer (%)	Calculated Molar Content of Gd_2_O_3_ in the Layer (%)
Point 1	Zr	22.45	5.57	5	4.54
	Gd	3.66	0.53		
Point 2	Zr	22.98	5.82	5	6.58
	Gd	5.55	0.82		

**Table 2:** Average chemical composition carried out by EDS point analysis at points 1 and 2 presented in Figure 2a.

Point Number	Element	Weight (%)	Atomic (%)	Expected Molar Content of Gd_2_O_3_ in the Layer (%)	Calculated Molar Content of Gd_2_O_3_ in the Layer (%)
Point 1	Zr	30.51	10.92	10	15.08
	Gd	18.69	3.88		
Point 2	Zr	31.97	11.82	10	11.92
	Gd	14.93	3.20		

**Table 3:** Average chemical composition performed by EDS point analysis at point 1 shown in Figure 6a.

Point Number	Element	Weight (%)	Atomic (%)	Expected Molar Content of Gd_2_O_3_ in the Layer (%)	Calculated Molar Content of Gd_2_O_3_ in the Layer (%)
Point 1	Zr	31.35	8.81	5	5.37
	Gd	6.15	1.00		

## Data Availability

The data presented in this study are available upon request from the corresponding author.
